# Impact of Anabolic Steroids on Tendons: A Narrative Review

**DOI:** 10.7759/cureus.93649

**Published:** 2025-10-01

**Authors:** Wojciech Żywiec, Alicja Dorota, Karol Kozłowski, Michał Dorota, Cezary Milczarek, Illia Koval, Anna Mariankowska, Bartosz Czyżewski, Joanna Czyżewska, Nicole Maryniak

**Affiliations:** 1 Medicine, Provincial Hospital of St. Luke in Tarnów, Tarnów, POL; 2 Medicine, Zaglebiowskie Oncology Center in Dąbrowa Górnicza, Dąbrowa Górnicza, POL; 3 Anesthesia and Critical Care, Provincial Integrated Hospital in Kielce, Kielce, POL; 4 Conservative Dentistry, Central Clinical Hospital of the Medical University of Lodz, Łódź, POL; 5 Medicine, Central Clinical Hospital of the Medical University of Lodz, Łódź, POL; 6 Medicine, Provincial Hospital in Poznań, Poznań, POL; 7 Rheumatology, Central Clinical Hospital of the Medical University of Lodz, Łódź, POL

**Keywords:** anabolic-androgenic steroids, doping, injury risk, musculoskeletal injuries, overuse injuries, side effects, strength training, tendon rupture, testosterone

## Abstract

Anabolic-androgenic steroids (AAS), synthetic testosterone derivatives, are used to increase muscle mass and performance. However, high doses negatively affect tendons and ligaments, disrupting collagen balance and increasing injury risk due to a mismatch between muscle strength and tendon resilience. AAS use increases tendon rupture risk by weakening collagen structure and elasticity, despite muscle growth. Injuries often occur at major tendon sites and require imaging-based diagnosis, with surgery preferred in active individuals. Education and prevention are key due to AAS-related systemic risks. This review compiles data from clinical, experimental, and imaging studies on AAS effects on tendons. It focuses on structural changes, common rupture sites (e.g., triceps, pectoralis major, quadriceps), diagnostic tools, and treatment strategies. This review aims to analyze the effects of AAS on tendon structure and function, identify the most common tendon injuries associated with AAS use, and present current diagnostic and treatment strategies to aid in prevention and clinical management.

## Introduction and background

Anabolic-androgenic steroids (AAS) are synthetic, pharmacologically optimized analogues of testosterone, engineered to amplify its anabolic and androgenic properties [1]. These compounds function as ligands for intracellular androgen receptors (ARs), modulating transcriptional activity to promote skeletal muscle hypertrophy, enhance recovery, and augment physical performance. Their clinical and nontherapeutic applications are based on their capacity to upregulate protein synthesis, inhibit proteolysis, and stimulate erythropoiesis; however, their misuse in athletic contexts is associated with significant musculoskeletal morbidity [1,2]. 

Mechanistic pathways of action of genomic signaling via ARs

AAS passively diffuse across cell membranes and bind with high affinity to cytosolic ARs, initiating receptor dimerization and nuclear translocation [3-5]. The ligand-receptor complex binds androgen response elements in promoter regions of target genes, activating transcription of anabolic mediators (e.g., insulin-like growth factor 1 (IGF-1)) and suppressing myostatin, a negative regulator of muscle growth [6,7]. 

Hypertrophic and anticatabolic effects

AAS enhance nitrogen retention and ribosomal biogenesis, elevating the rate of myofibrillar protein synthesis. Concurrently, they antagonize glucocorticoid receptor signaling, mitigating muscle catabolism under conditions of metabolic stress (e.g., intense training) [1,8,9].

Tendon Pathophysiology and Structural Compromise Collagen Dysregulation

AAS disrupt the synthesis and cross-linking of collagen, the primary structural component of tendons [10]. 

Biomechanical Disparity

While AAS-induced muscle hypertrophy increases contractile force, tendons exhibit maladaptive responses, including reduced elasticity and diminished load tolerance. This creates a biomechanical mismatch, predisposing tendons to rupture under supraphysiological loads [11,12]. 

Synthesis and implications

The therapeutic promise of AAS is eclipsed by their propensity to induce profound musculoskeletal pathology. The pathophysiological dichotomy between rapid muscle strengthening and delayed tendon remodeling underscores a critical vulnerability in AAS users. Furthermore, their systemic endocrine disruption and propensity to promote high-risk behaviors exacerbate injury susceptibility. Current evidence advocates for multidisciplinary interventions, encompassing biomechanical education, hormonal monitoring, and collagen-targeted therapies to mitigate these risks in athletic populations. 

## Review

Methodology

This narrative review synthesizes current knowledge on AAS use, focusing on epidemiology, tendon and ligament injury mechanisms, diagnostics, treatment, and systemic adverse effects. Relevant experimental and clinical studies, including case reports and cohort studies, were analyzed thematically. Older studies were also included when they provided unique experimental insights, particularly on tendon structure and biomechanics, which remain relevant due to the limited number of investigations in this field. This approach provides a coherent overview of the topic and identifies areas for future research.

Epidemiology 

AAS use is highly prevalent among individuals engaged in resistance training. Among the 3,603 participants surveyed, 53.05% of men and 41.99% of women reported using AAS. Testosterone was the most commonly used substance among men (29.47%), while Winstrol was the most prevalent among women (31.20%). Additionally, 50.30% of male users administered steroids via injection, whereas 49.05% of female users preferred oral intake. Most participants had between six and 12 months of training experience, and 64.25% trained three times per week [13]. 

In Curitiba, Brazil, the use of anabolic steroids among gym goers is a notable concern. In a survey of 5,773 individuals across 100 gyms, 3.4% reported current AS use, 9.1% had used them in the past, and 4.3% intended to use them in the future. Men were significantly more likely to use AS, with 16.9% being current or former users, compared to 6.5% of women. The likelihood of AS use was higher among individuals aged 18-44. Additionally, longer training duration correlated with increased AS use, while beginners showed little interest in these substances [14]. 

The lifetime prevalence of AAS use among gym users in the United States is estimated at 3.0%. This figure is slightly below the global average of 3.3% but aligns with trends observed in other Western countries. Notably, AAS use is significantly more prevalent among men (6.4%) compared to women (1.6%). The study also highlights that recreational athletes exhibit the highest prevalence rates (18.4%), surpassing those of elite athletes (13.4%) and prisoners (12.4%). These findings suggest that AAS use extends beyond professional sports, permeating into the general population, particularly among individuals engaged in resistance training for aesthetic or personal goals [15,16]. 

The overall prevalence of AAS use among Swedish adolescents was reported at 2.9% for boys and 0.0% for girls. The study also found that AAS use was significantly associated with other risk behaviors, such as use of narcotics, high alcohol consumption, and participation in violent acts [17]. 

Injury mechanisms 

The most common mechanisms underlying tendon injuries in AAS users (Table 1) include disruption of the extracellular matrix through collagen fibril dysplasia, which weakens tendon architecture and reduces viscoelastic properties [18,19]. 

**Table 1:** Structural and biomechanical changes in tendons induced by AAS AAS: anabolic-androgenic steroids

Parameter	Changes due to AAS	Clinical consequences
Collagen	Disrupted synthesis and cross-linking	Weakened tendon architecture
Matrix metalloproteinases (MMP-2)	Decreased activity	Impaired extracellular matrix remodeling
Paratenon	Thickening	Fibrosis, inflammatory infiltrates
Biomechanical properties	Reduced elasticity, increased stiffness	Increased susceptibility to rupture, microdamage
Tendon compliance	Decreased	Reduced flexibility and energy storage capacity

Steroid-induced downregulation of matrix metalloproteinases and impaired collagen turnover further compromise tendon remodeling and repair, especially under mechanical load. These changes lead to decreased tendon compliance and flexibility, predisposing tendons to microdamage, inflammation, fibrosis, and ultimately increased risk of acute ruptures, often on a background of pre-existing degenerative tendinopathy exacerbated by high mechanical stress [10]. 

Administration of AAS in rats, both with and without concurrent exercise, significantly alters structural and molecular characteristics of the Achilles tendon. AAS use leads to increased collagen fiber aggregation, thickening of the paratenon, and reduced activity of matrix metalloproteinase-2, an enzyme essential for extracellular matrix remodeling. When combined with resistance training, these changes are accompanied by inflammatory infiltrates and fibrosis, indicating impaired adaptive responses of tendons to mechanical loading due to disrupted collagen turnover and repair mechanisms [10,20]. 

Male weightlifters with acute total pectoralis major muscle (PMM) tendon ruptures often exhibit signs of tendinopathy on magnetic resonance imaging (MRI) in contralateral tendons, with histological analysis confirming degenerative changes in over 66% of ruptured tendons. These data suggest that acute PMM tendon rupture is frequently preceded by pre-existing tendinous degeneration, potentially exacerbated by anabolic steroid use and high mechanical loads during resistance training [21]. 

Nandrolone decanoate administration combined with mechanical loading reduces tendon compliance in various rat tendons. The calcaneal tendon exhibits an extended toe region, increased resistance to tensile load, and decreased elastic modulus, features indicative of altered energy storage capacity. These effects are amplified by exercise, particularly in the deep flexor tendon, which plays a key role in rapid force transmission during jumping. This evidence supports the hypothesis that anabolic steroid use, especially combined with mechanical loading, decreases tendon flexibility and may increase the risk of tendon rupture [22]. 

Most common injury locations 

*Distal Triceps Tendon* 

The distal triceps tendon is the conjoined insertion of the long, lateral, and medial heads of the triceps brachii onto the olecranon, forming a robust, broad tendinous structure. Despite its strength, rupture of the distal triceps tendon remains rare, representing less than 1% of all tendon injuries in the upper extremity and typically affecting males aged 30 to 50 years. Clinically, patients may present with posterior elbow pain, swelling, ecchymosis, and weakened active extension. Complete ruptures, while dramatic, may retain partial extension due to intact lateral expansions [23]. Mechanisms of injury often involve eccentric loading or sudden resisted elbow extension, commonly during activities such as weightlifting or after a fall on an outstretched hand [24]. An important contributing factor is the use of AAS, which has been associated with either spontaneous or minimally traumatic ruptures [25,26]. 

Pectoralis Major 

The PMM, consisting of clavicular and sternocostal heads, is critical for shoulder adduction and internal rotation. Ruptures of this muscle are more prevalent among physically active males, particularly during activities involving high eccentric loading, such as bench pressing. Injuries most frequently occur at the tendinous insertion on the humerus [27]. Clinical symptoms include acute pain, ecchymosis, weakness in shoulder function, and a noticeable deformity of the anterior axillary fold, often requiring an MRI for accurate diagnosis and classification. A notable risk factor in this patient population is the use of AAS. Histopathological evidence and clinical correlation suggest that AAS compromise tendon integrity, which predisposes to rupture under physiologic or submaximal loads [27-31]. 

*Quadriceps * 

The quadriceps tendon, responsible for transmitting force from the quadriceps muscle group to the patella to facilitate knee extension. Quadriceps tendon rupture (QTR) is an uncommon, yet debilitating injury, most common in middle-aged men, often associated with underlying risk factors such as AAS use. Clinically, QTR presents with sudden anterior knee pain, swelling, and loss of active knee extension, necessitating prompt diagnosis and surgical repair to restore function. Bilateral ruptures, while rare, have been reported with increasing frequency in individuals engaging in high-intensity resistance training and AAS consumption, suggesting a causal link between steroid-induced tendon degeneration and rupture susceptibility [32,33]. 

*Ligaments of the Knee Area* 

The knee joint stability is maintained by a complex ligamentous system consisting primarily of the cruciate ligaments: the anterior cruciate ligament (ACL), posterior cruciate ligament (PCL), and the collateral ligaments, including the medial collateral ligament (MCL) and lateral collateral ligament (LCL). The ACL controls anterior translation and rotational stability of the tibia relative to the femur, whereas the PCL prevents posterior displacement of the tibia. The MCL resists valgus stress and the LCL counteracts varus forces, both contributing significantly to mediolateral knee stability. Furthermore, extracapsular ligaments such as the oblique and arcuate popliteal ligaments reinforce the posterior and posterolateral knee capsule, enhancing overall joint stability [34]. AAS use has been associated with deleterious effects on ligamentous tissue, which may predispose ligaments to injury under physiological loads. Such structural changes in ligaments can increase the risk of tears, particularly among individuals engaged in high-intensity physical training and AAS consumption. However, direct clinical evidence linking AAS use to knee ligament ruptures remains limited and requires further investigation [32,35]. 

Diagnostics 

**Figure 1:**
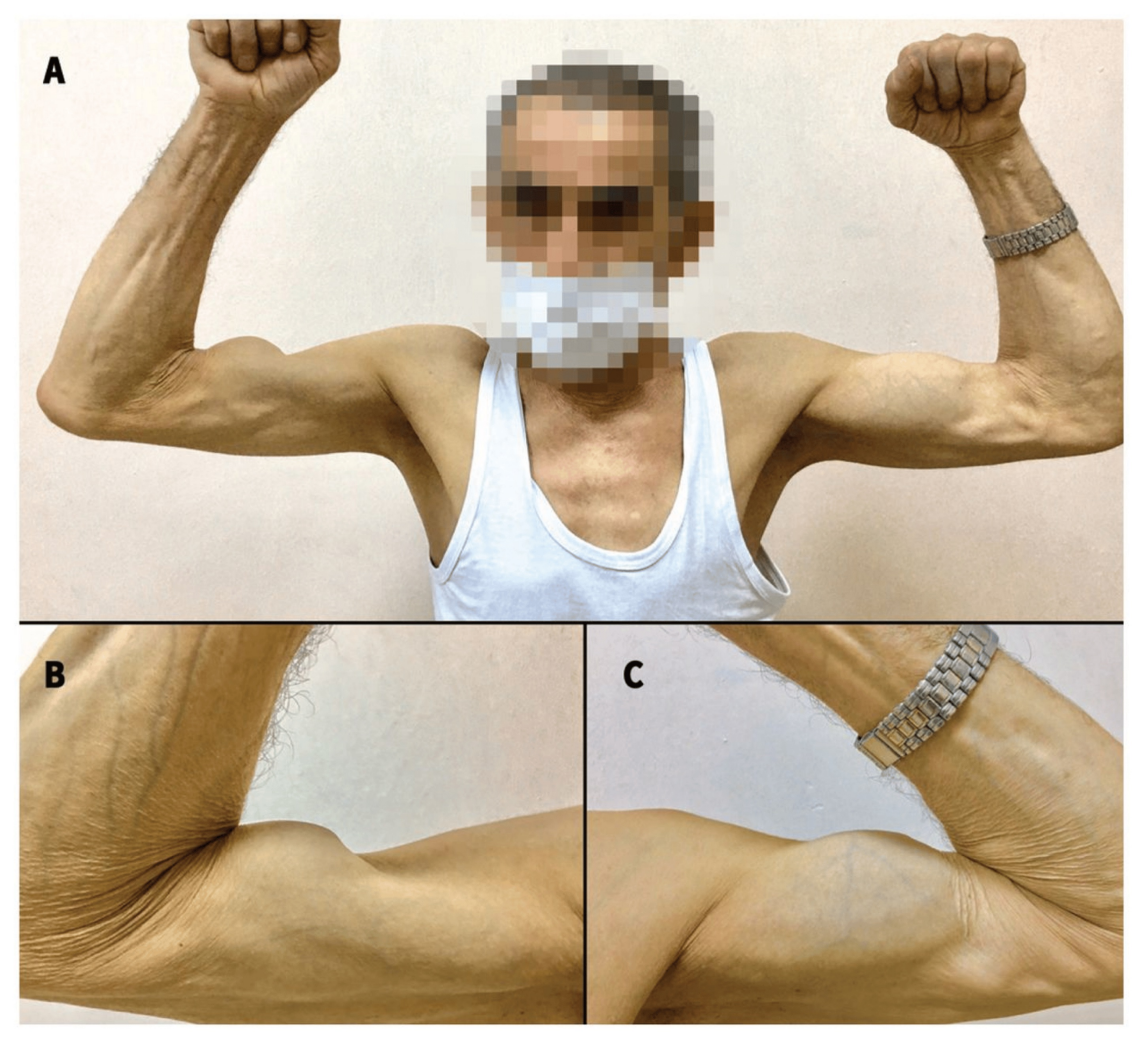
"Popeye sign”-proximal biceps tendon rupture in a 70-year-old male patient Adapted/reproduced from Kayaalp and Cirdi [36], with permission

Diagnosis of complete and partial tendon ruptures is based on symptoms presented and reported by patients and primarily on imaging studies. During the physical examination, key findings include acute, stabbing pain at the site of injury, exacerbated by palpation or attempted active muscle contraction, along with swelling, ecchymosis, muscle weakness, functional deficit in the range of motion dependent on the injured tendon, limitation of range of motion, and joint stiffness. For complete ruptures, characteristic features include an audible "snap" at the moment of injury, anatomical deformity, a visible gap in the tendon continuity, proximal retraction of the muscle tissue forming a mass, complete loss of function, absence of muscle tension during resistance testing, for example, the "Popeye sign” (Figure 1), manifesting as proximal displacement of the muscle belly [23,32,33]. 

In imaging diagnostics, radiographic images were historically performed; however, the current standard relies on significantly more accurate soft tissue imaging modalities, primarily MRI, but also ultrasonography (US) and computed tomography (CT). These techniques enable differentiation between acute and chronic changes. Both US and MRI facilitate the distinction between partial and complete tendon ruptures. MRI is the diagnostic modality of choice for evaluating ligamentous injuries, facilitating accurate assessment of lesion extent and guiding appropriate management strategies [21,23,24,27]. 

**Figure 2:**
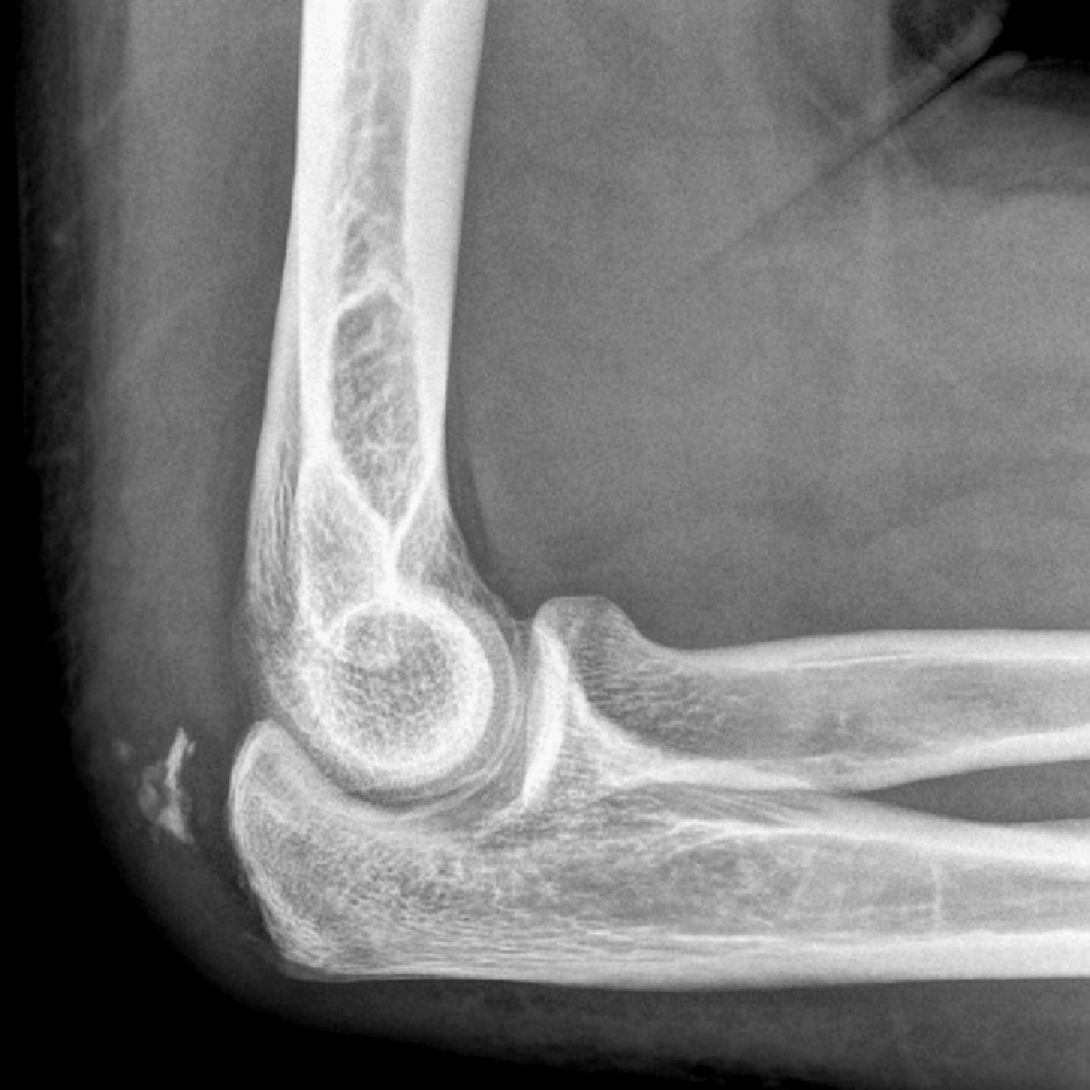
"Flake of bone"-typical sign on lateral X-ray image for triceps avulsion injuries Adapted/reproduced from Geyer et al. [37], with permission

One of the more frequent injuries associated with anabolic steroid use is triceps tendon rupture, for which a notable and diagnostically useful radiological finding is the presence of a small avulsion fracture from the olecranon process called the "Flake sign" (Figure 2). While MRI provides a definitive assessment of the tear extent and retraction, this represents a pathognomonic sign for this injury [28]. 

Accurate imaging of each injury allows for the correct classification of the severity grade and enables the proposal of the optimal treatment approach for the individual patient. 

Treatment 

Treatment of tendon ruptures is analogous across the most common anatomical locations associated with this type of injury. The most significant classification distinguishes between conservative and surgical management. Generally, partial tears presenting with minimal symptoms can be managed nonsurgically, whereas complete ruptures are typically treated with surgical repair. 

Conservative Treatment 

Conservative management is usually less effective and more frequently results in incomplete restoration of strength or range of motion [38]. Nonetheless, it remains the sole option in certain cases. This is particularly relevant for patients with medical comorbidities, advanced age, incomplete tears, or irreparable damage. Patients undergoing delayed repair have demonstrated significantly better outcomes in terms of strength, satisfaction, and overall function compared to those receiving nonoperative treatment following pectoralis major rupture. Conservative treatment often involves a period (generally 3-4 weeks) of immobilization in flexion, followed by gradual flexion mobilization [28]. 

Surgical Treatment 

An important factor influencing the decision for surgical intervention is the patient's young age, as surgical repair tends to provide a superior prognosis regarding the restoration of full muscular strength and return to physical activity, especially in athletes [39]. Tendon ruptures can be categorized as acute or chronic; however, treatment protocols are generally guided by the same principles regardless of the timing of injury. While chronicity may influence the choice of surgical technique, it should not preclude repair [40]. Various techniques for tendon repair have been described, with no single method demonstrating clear superiority in terms of clinical outcomes; each offers theoretical advantages [41]. 

Adverse effects 

The utilization of AAS constitutes a substantial concern, extending beyond tendon ruptures. Although not the primary focus of this article, it is imperative to succinctly address the numerous adverse effects (Table 2) associated with this form of doping. 

**Table 2:** Systemic adverse effects of AAS AAS: anabolic-androgenic steroids; LH: luteinizing hormone; FSH: follicle-stimulating hormone

Organ system	Effects/changes	Reversibility
Cardiovascular	Hypertension, left ventricular hypertrophy, myocardial infarction	Partially irreversible
Renal	Rhabdomyolysis, acute renal failure	Potentially reversible, depending on the damage
Endocrine (males)	↓ LH, ↓ FSH, testicular atrophy, impaired spermatogenesis	Partially reversible
Endocrine (females)	Hirsutism, acne, deepening of voice, clitoral hypertrophy, male-pattern baldness	Often irreversible
Psychiatric	Aggressiveness, depression, euphoria, mood swings, altered libido, psychosis	Reversible, but risk of long-term changes
Growth (adolescents)	Premature epiphyseal closure	Irreversible

Premature epiphyseal closure in children and adolescents represents a significant consequence, resulting in a decrease in adult height, following prolonged androgen exposure [42]. Hypertension is correlated with AAS use, and myocardial infarction has been documented among athletes engaging in prolonged steroid administration [43]. 

Furthermore, AAS use is linked to irreversible myocardial alterations, including concentric left ventricular hypertrophy [43,44]. Psychiatric manifestations may occur, encompassing irritability, aggressiveness, euphoria, depression, mood swings, altered libido, and psychosis [45]. Acute renal failure secondary to rhabdomyolysis in AAS-using bodybuilders has been reported. Concurrent abuse of AAS poses a significant risk for renal damage [46]. 

In males, AAS suppresses luteinizing hormone (LH) and follicle-stimulating hormone (FSH) levels, which leads to decreased endogenous testosterone production, impaired spermatogenesis, and testicular atrophy [47]. Gynecomastia may arise from peripheral androgen conversion to estradiol and estrone. This effect may be markedly exacerbated in individuals with liver disease, likely attributable to reduced hepatic clearance of the parent steroid or its estrogenic metabolites [48]. 

In females, AAS use extends beyond menstrual abnormalities to include pronounced masculinizing effects: hirsutism, acne, vocal deepening, clitoral hypertrophy, and male-pattern baldness. Certain of these androgenic effects may be persistent or irreversible [49]. 

Limitations

Limited Clinical Evidence

While numerous animal studies demonstrate deleterious structural and molecular changes in tendons following AAS exposure, human clinical evidence remains relatively scarce. Most available data are derived from case reports, small cohort studies, or retrospective analyses, which limit generalizability.

Confounding Variables

Many AAS users simultaneously engage in high-intensity resistance training, polypharmacy (stacking of multiple substances), or concomitant recreational drug use. These factors make it difficult to isolate the independent effects of AAS on musculoskeletal integrity.

Lack of Longitudinal Data

Few prospective studies follow athletes over extended periods, resulting in insufficient understanding of the long-term progression of tendon and ligament degeneration associated with chronic AAS exposure.

Heterogeneity of AAS Compounds and Dosing

The wide range of synthetic derivatives, administration routes (oral vs. injectable), dosing regimens, and cycling strategies complicates the interpretation of findings and their clinical translation.

Recommendations

Athletes and recreational users suspected of AAS exposure should undergo routine musculoskeletal assessments, including imaging of high-risk tendons (triceps, pectoralis major, quadriceps, Achilles) to detect early degenerative changes. Treatment strategies should integrate orthopedic, endocrinological, cardiological, and psychiatric evaluation to address the systemic and musculoskeletal consequences of AAS use. Prevention should focus on public health strategies, targeting gyms, athletic organizations, and young populations, emphasizing the risks of tendon rupture, cardiovascular complications, and endocrine dysfunction. Post-injury rehabilitation in AAS users should account for impaired collagen turnover and altered tendon healing capacity, potentially requiring modified loading strategies and longer recovery periods compared to nonusers.

Future directions

Large-scale, long-term studies are necessary to clarify the causal relationship between AAS exposure and tendon/ligament injuries, particularly in populations engaging in resistance training. Advanced imaging, histopathology, and molecular analyses should be employed to better define the specific pathways by which AAS disrupt collagen organization and tendon biomechanics. Future work should differentiate between the effects of various AAS derivatives, dosing regimens, and modes of administration on tendon health. Research should investigate whether pharmacological or biological therapies (e.g., collagen-targeted agents, growth factor modulation, regenerative medicine approaches) can mitigate the detrimental musculoskeletal consequences of AAS. Findings should inform anti-doping policies, injury prevention programs, and rehabilitation guidelines tailored for populations at risk of AAS-associated musculoskeletal injuries.

## Conclusions

AAS, while effective in enhancing muscle mass and physical performance, pose significant risks to musculoskeletal integrity, particularly tendinous structures. The biomechanical imbalance induced by rapid muscle hypertrophy without concurrent tendon adaptation leads to increased susceptibility, tendon degeneration, microtrauma, and rupture. Epidemiological data confirm that AAS use is prevalent not only among elite athletes but also within the general population. This widespread misuse is concerning, given the clear association with severe tendon injuries, particularly in areas subjected to intense eccentric loading, such as the distal triceps tendon, pectoralis major, quadriceps tendon, and potentially the ligaments of the knee. Diagnosis of tendon ruptures relies heavily on imaging modalities, particularly MRI and US. Management approaches vary by severity, ranging from conservative treatment in minor tears to surgical intervention in complete or functionally significant ruptures. Beyond musculoskeletal implications, AAS use is associated with a host of systemic adverse effects, including cardiovascular, endocrine, psychiatric, renal, and reproductive complications. These multifaceted risks underscore the need for comprehensive public health strategies targeting AAS misuse, including preventive education, harm reduction protocols, and multidisciplinary management of affected individuals. 

In sum, while anabolic steroids may offer short-term performance enhancement, their long-term impact on tendon health and systemic physiology renders them a high-risk intervention. Future efforts should focus on increasing awareness, refining diagnostic tools, and optimizing therapeutic approaches to mitigate the consequences of AAS-related musculoskeletal injuries.
